# Techno‐Economic Assessment of a Hydrogen‐Assisted Hybrid Renewable Microgrid with Fuel Cells for Off‐Grid Electrification

**DOI:** 10.1002/gch2.70122

**Published:** 2026-06-08

**Authors:** Taib Hasan, Prapti Das, Md. Feroz Ali, Md Shafiul Alam, Mohammad Ali, Imil Hamda Imran

**Affiliations:** ^1^ Department of Electrical and Electronic Engineering Pabna University of Science and Technology Pabna Bangladesh; ^2^ Department of Electrical Engineering College of Engineering King Faisal University Al Ahsa Saudi Arabia

**Keywords:** HOMER Pro, hybrid renewable energy system, hydrogen storage, off‐grid microgrid, sensitivity analysis

## Abstract

Rural electrification in coastal Bangladesh faces challenges from geographic isolation, weak grid access, and growing energy demand. This study designs and evaluates an off‐grid hybrid renewable microgrid for Char Ishwar, Noakhali, using HOMER Pro (version 3.14.2). The proposed system integrates solar photovoltaic (PV), wind turbine (WT), battery energy storage system (BESS), electrolyzer, hydrogen (H_2_) storage tank, and fuel cell (FC) to enhance reliability and mitigate renewable intermittency. Three configurations were analyzed: Case A (PV–WT–BESS–Converter–Electrolyzer–FC–H_2_ Tank), Case B (WT‐based), and Case C (PV‐based), supplying electricity to 180 rural households. Case A emerged as the optimal solution, achieving a cost of energy (COE) of $0.139/kWh, a net present cost (NPC) of $1.28 million, and a capital cost of $594,206. The system achieved near‐zero net CO_2_ emissions of –3.79 kg/year, a value arising from HOMER Pro's baseline emission offset accounting, wherein avoided emissions from near‐100% renewable generation are credited against the assumed grid emission factor, rather than implying physical carbon sequestration, highlighting the substantial environmental benefits of green hydrogen integration. Comprehensive sensitivity analysis shows wind speed and load demand strongly influence system economics, validating configuration robustness and enabling sustainable, reliable, cost‐effective electrification for coastal Bangladesh.

## Graphical abstract

This study presents a hydrogen‐assisted hybrid renewable microgrid optimized using HOMER Pro for off‐grid electrification in coastal Bangladesh. Integrating solar PV, wind, BESS, electrolyzer, hydrogen storage, and fuel cells, the system achieves a low cost of energy ($0.139/kWh), high reliability, and near‐zero emissions, offering a sustainable and resilient solution for remote communities.

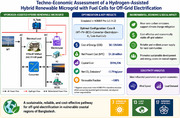

**Abbreviations**
BESS: Battery Energy Storage System
CDF: Cumulative Distribution Function
COE: Cost of Energy
DC: Duration Curve
DSM: Demand‐Side Management
FC: Fuel Cell
GHI: Global Horizontal Irradiation
HOMER: Hybrid Optimization of Multiple Energy Resources
HRES: Hybrid Renewable Energy System
IDCOL: Infrastructure Development Company Limited
LCOE: Levelized Cost of Energy
LCOH: Levelized Cost of Hydrogen
NASA: National Aeronautics and Space Administration
NPC: Net Present Cost
NREL: National Renewable Energy Laboratory
NSGA‐II: Non‐dominated Sorting Genetic Algorithm II
P2G2P: Power‐to‐Gas‐to‐Power
PBTM: Payback Time in Monetary Terms
PV: Photovoltaic
RF: Renewable Fraction
SOC: State of Charge
SSE: Surface Meteorology and Solar Energy
WT: Wind Turbine

## 1 Introduction

Amid rising global energy demand and the urgent necessity to address climate change, the pursuit of sustainable and renewable energy solutions has become increasingly vital. Bangladesh, as a developing nation with a rapidly growing population, faces a severe energy crisis that poses risks to both socioeconomic progress and environmental sustainability [1]. With more than 160 million inhabitants and a steadily expanding economy, the country's energy requirements have grown more complex and demanding [2, 3]. Recent global assessments indicate that nearly 675 million people worldwide remain without electricity, including about 41 million in Bangladesh, which continues to hinder productivity and sustainable development [4]. The nation's energy insecurity is made worse by its reliance on nonrenewable energy sources, which also contribute to environmental damage by emitting large amounts of CO_2_ [5]. Currently, a significant share of global energy demand is met through fossil fuels, which are major contributors to global warming [6]. Hydrogen, owing to its high specific energy, has emerged as a promising energy carrier and an efficient medium for energy storage [7]. While hydrogen can be derived from resources such as coal, natural gas, and biomass, its production through water electrolysis powered by renewable energy sources has demonstrated considerable potential in minimizing environmental impacts. Renewable energy systems can address the limitations of fossil fuels by fulfilling energy demand, enabling long‐term sustainable generation, and reducing adverse environmental consequences [8]. However, fluctuations in renewable energy output, driven by the variable nature of weather conditions such as solar radiation and wind speed, create reliability challenges. To overcome these issues, storage systems become indispensable, though often associated with high costs. In off‐grid renewable applications, the limited capacity of conventional storage frequently leads to excess energy, which in some cases may surpass 60% of total generation [9]. Integrating a hydrogen production subsystem offers a practical solution by converting surplus energy into green hydrogen via an electrolyzer, with storage in hydrogen tanks for later utilization [10]. This stored hydrogen can subsequently be converted back into electricity using FCs, which act as backup units to meet demand during periods of low renewable generation. Consequently, hydrogen applications in renewable‐based systems hold significant promise for electrification in remote and underserved regions [11]. Renewable energy technologies are increasingly recognized as sustainable alternatives to fossil fuels. As of 2026, Bangladesh's total installed renewable capacity reaches 1,694.46 MW, with grid‐connected systems contributing 77.7% and off‐grid systems 22.3%. Solar energy dominates the mix (82.7%), followed by hydropower (13.6%) and wind (3.7%), while biomass and biogas play minor roles [12]. Around six million Solar Home Systems deployed under the IDCOL program highlight the success of off‐grid electrification [13, 14]. However, large‐scale grid integration remains constrained by financial, infrastructural, and technical challenges. To address these issues, this study proposes a hybrid renewable microgrid for Char Ishwar, Noakhali, integrating solar PV, WTs, battery storage, hydrogen production and storage, and FCs. Optimized using HOMER Pro, the system ensures reliable, low‐carbon electricity for remote coastal communities.

Hybrid renewable–hydrogen microgrids have gained significant scholarly attention as viable solutions for low‐carbon, resilient, and decentralized energy systems, particularly in regions with high fuel costs, unreliable grids, or strong decarbonization goals. A consistent finding across the literature is the trade‐off between environmental benefits and the high capital and storage costs of hydrogen technologies, which continue to limit large‐scale deployment despite technological progress. Many studies emphasize optimal sizing and techno‐economic performance under diverse climatic and load conditions. Alturki (2022) [15] optimized a PV–wind–generator–storage–FC system in Yanbu, Saudi Arabia, achieving an LCOE of $0.155/kWh and an LCOH of $25.6/kg, while identifying hydrogen components as the dominant contributor to NPC. Similarly, Alharthi (2024) [16], showed that insufficient battery integration reduces FC lifetime and increases costs, highlighting the need for hybrid storage architectures. Regional comparisons further demonstrate context‐dependent feasibility. Ram et al. (2023) [17] reported lower costs in Fiji due to complementary renewable resources, whereas Kumar et al. (2024) [18] emphasized the role of financial incentives in rural India. DSM has been shown to improve system economics, as demonstrated by Youssef et al. (2024) [19], though excess electricity and storage inefficiencies remain challenges. Overall, the literature underscores that integrated storage design, optimized dispatch, and supportive policies are essential for advancing hydrogen‐based microgrids. Tiong et al. (2022) [20] demonstrated that grid‐connected microgrids integrating solar, wind, battery, and hydrogen storage achieve lower NPC and LCOE than stand‐alone systems, though reliance on grid electricity reduces overall emission mitigation, highlighting a trade‐off between cost efficiency and decarbonization depth. In an urban European context, Basnet et al. (2024) [21] showed that at electricity prices above €140/MWh, renewable sources could supply up to 70% of grid demand and 80% of electrolyzer demand, substantially lowering CO_2_ emissions, yet hydrogen production costs remained high, emphasizing the need for policy support and electrolysis cost reductions. Comparative storage studies consistently identify hybrid solutions as cost‐optimal. El Bakkali et al. (2022) [22] found that combined battery–hydrogen systems minimized energy costs, whereas hydrogen‐only storage resulted in prohibitive NPC. Similarly, Kapen et al. (2022) [23] reported very low LCOH under optimized hybrid configurations despite high upfront investments. Sector‐specific analyses further highlight hydrogen's flexibility benefits. Vichos et al. (2022) [24] reported LCOE reductions exceeding 40% in port applications, while Cruz‐Soto et al. (2022) [25] emphasized the need for major FC cost reductions. Advanced optimization studies by Akarsu et al. (2022) [26] and Le et al. (2023) [27] underscore the role of sophisticated algorithms in balancing cost, reliability, and emissions. Large‐scale analysis by Timalsina and Ghahremanlou (2024) [28] optimized a wind‐to‐hydrogen system in Newfoundland and achieved a short‐term LCOH of $3.43/kg, illustrating the significant cost advantages achievable at scale. Song et al. (2024) [29] investigated 100% renewable energy systems for data centers in South Korea and projected that on‐site hydrogen production becomes economically viable after 2030, with NPC reductions exceeding 75% as green hydrogen prices decline, highlighting the strong time dependence of hydrogen competitiveness. Review studies provide broader system‐level insights. Modu et al. (2023) [30] reported efficiency improvements of up to 85% and cost reductions of 20% in hybrid renewable–hydrogen systems, while identifying high capital investment and storage losses as persistent barriers. Li et al. (2023) [31] showed that coordinated electric and hydrogen storage across multiple microgrids can reduce costs by 15% and enhance reliability by 25%, although hydrogen storage remains the dominant cost component. Yousri et al. (2023) [32] demonstrated that advanced energy management systems can deliver customer savings exceeding 80%, albeit with increased system complexity. Application‐specific studies confirm hydrogen's versatility but reiterate economic challenges. Urs et al. (2023) [33] achieved a 78% RF with an LCOH of $3.117/kg in a UAE commercial microgrid. Optimization studies by Kumar et al. (2022) [34], Alturki (2022) [15], Mandić et al. (2023) [35], and Caliskan and Percin (2024) [36] consistently show that hydrogen enhances long‐duration storage and reliability under optimized hybrid configurations. Alhawsawi et al. (2024) [37] performed a comparative assessment of geothermal, wind, and photovoltaic resources, concluding that PV‐grid‐connected hybrid systems deliver the highest energy production with minimal system complexity. Bin et al. (2022) [38] demonstrated that optimal battery energy storage sizing in campus microgrids reduced grid electricity costs by 36.6%, yielding savings of $668.8/day, though grid dependency persisted as a limitation. Environmental performance remains a key driver of recent microgrid research. Almohaimeed et al. (2023) [39] quantified CO_2_ emission reductions at Qassim University from 615.8 Mt to 147.4 Mt following microgrid deployment, while Abdelsattar et al. (2024) [40] reported concurrent reductions in emissions and operational costs in Hurghada, Egypt, using PV–wind–diesel hybrids. Islanded and rural electrification studies further highlight the role of HRES in energy access. Molu et al. (2023) [41] demonstrated that a solar–wind–biogas hybrid system for Manoka Island, Cameroon, achieved an energy cost of $0.1981/kWh and an NPV of $2 209 741, though high battery costs remained a challenge. Pérez Uc et al. (2024) [42], in a comprehensive review of HOMER‐based HRES applications, confirmed that integrating solar, wind, and biomass reduces fossil‐fuel dependence across sectors, but high capital costs and technological constraints persist. Barakat et al. (2024) [43] showed that advanced multi‐objective optimization using NSGA‐II significantly improves the economic and sustainability performance of PV–wind–battery systems with EV charging integration, emphasizing the importance of algorithmic advances alongside hardware cost reductions. Mohamed G. Moh Almihat and Kahn (2023) [44] emphasized that integrated control strategies are essential to minimize COE and NPC in solar–wind–diesel–battery microgrids. Demand‐side efficiency measures increasingly complement supply‐side optimization. Nassar et al. (2024) [45] showed that replacing conventional lighting with LEDs reduced required batteries from 56 to 22 and PV panels from 20 to 8, achieving an LCOE of $0.12/kWh and reducing NPC by 30%. Nassar et al. (2024) [46] demonstrated that optimized hybrid systems could supply 82% of Palestine's energy demand from renewables, significantly reducing costs and emissions. El‐Khozondar et al. (2022) [47] showed that PV–wind–diesel systems for COVID‐19 quarantine centers in Gaza reduced COE by 54.89% with a payback period of 1.8 years. Hakizimana et al. (2024) [48] demonstrated that grid‐connected PV systems in industrial facilities achieved a 10‐year payback and reduced CO_2_ emissions by 6,555.1 tons annually. Overall, the literature shows that hybrid renewable–hydrogen energy systems, optimized using HOMER Pro and advanced algorithms, can substantially reduce energy costs, emissions, and grid dependence. Grid‐connected systems generally offer better economic performance, while off‐grid systems achieve higher renewable fractions at higher costs. Hydrogen is most effective when combined with battery storage and advanced energy management. However, high capital costs, storage inefficiencies, and system complexity persist, highlighting the need for cost reductions, improved optimization, and supportive policy frameworks.

Despite the growing interest in hybrid renewable energy systems (HRES) in Bangladesh, existing studies are predominantly limited to single‐source or dual‐source configurations, such as standalone solar PV or wind‐based microgrids. The integrated deployment of advanced multi‐source systems combining solar PV, WTs, BESS, and hydrogen‐based technologies—including electrolyzers, hydrogen storage tanks, and FCs—remains insufficiently explored under realistic operating conditions. Moreover, prior research rarely incorporates long‐term techno‐economic optimization using high‐resolution time‐series data that reflect local climatic variability, seasonal demand fluctuations, and component‐level interactions. Comprehensive sensitivity analyses addressing uncertainties in renewable resource availability, load demand, and economic parameters are also scarce, leaving critical questions unresolved regarding the feasibility, optimal configuration, cost‐effectiveness, reliability, and emission reduction potential of hydrogen‐assisted hybrid microgrids for rural electrification in Bangladesh. To address these gaps, this study aims to design and evaluate an optimized off‐grid hybrid renewable microgrid tailored to rural coastal communities by integrating solar PV, WTs, BESS, electrolyzers, hydrogen tanks, and FCs. Using HOMER Pro, multiple system configurations are assessed to identify the optimal solution based on COE, NPC, system reliability, and environmental performance. The study further examines the role of hydrogen‐based long‐term storage in mitigating renewable intermittency and enhancing energy security, supported by extensive sensitivity analyses to evaluate system robustness under varying climatic, load, and economic conditions. Collectively, this work contributes a comprehensive techno‐economic framework, quantitative insights into hydrogen–battery hybrid storage integration, and policy‐relevant guidance for deploying scalable, low‐carbon microgrids in remote and vulnerable regions of Bangladesh.

## 2 Materials and Methods

### 2.1 Site Location

**FIGURE 1:**
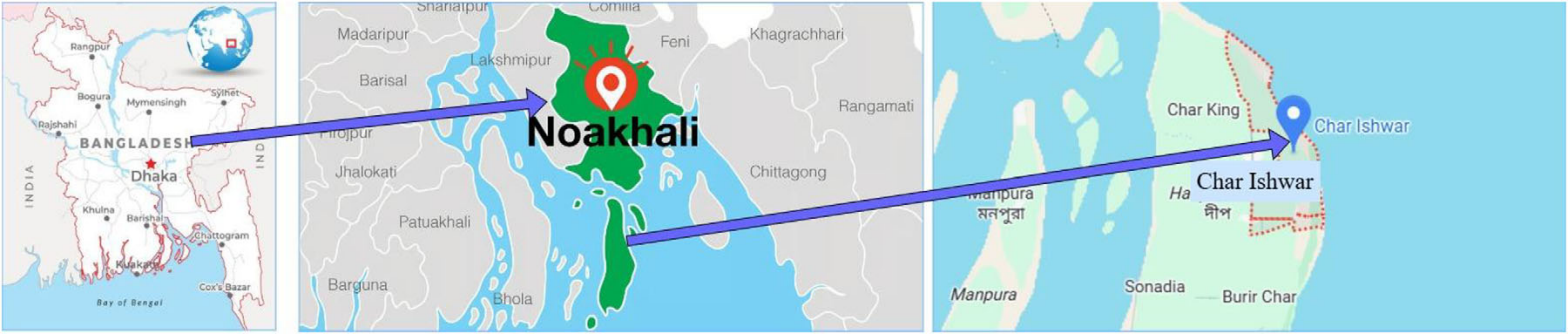
Geographic location of the study area.

Char Ishwar, situated in the eastern region of Hatiya Upazila under Noakhali District, Bangladesh (22°17.2′N, 91°10.0′E), covers an area of around 55 square kilometers. The proposed microgrid is designed to meet a daily community demand of 1956.87 kWh with a peak load of 268.19 kW. Being a coastal and riverine area, Char Ishwar faces high exposure to tidal surges, flooding, and erosion, while also offering prospects for agriculture, fisheries, and renewable energy deployment. Figure 1 highlights its geographical boundaries, underscoring the critical need for resilient energy systems and sustainable development measures in this vulnerable region.

### 2.2 Load Profile

Table 1 presents the estimated load calculation for a single household, based on common electrical appliances such as fans, lighting, TV, iron, desktop, refrigerator, random load, and water pump. The total daily consumption is 10.8715 kWh/day, where the refrigerator and water pump are the major contributors. This load represents a community‐type demand profile, typical of rural households. When scaled to 180 households, the community demand reaches 1956.87 kWh/day with a peak load of 268.19 kW.

**TABLE 1:** Daily Energy Consumption of Electrical Components.

Components	Ratings	Quantity	Operating hours per day	Total demand (kWh/d)
Fan	60	4	8	1.92
Lighting Bulbs	15	5	10	0.75
TV	50	1	5	0.25
Iron	1300	1	0.175	0.2275
Desktop	250	1	4	1
Refrigerator	350	1	13	4.55
Random Load	56	1	6	0.224
Water pump	1300	1	1.5	1.95
Total				10.8715

**FIGURE 2:**
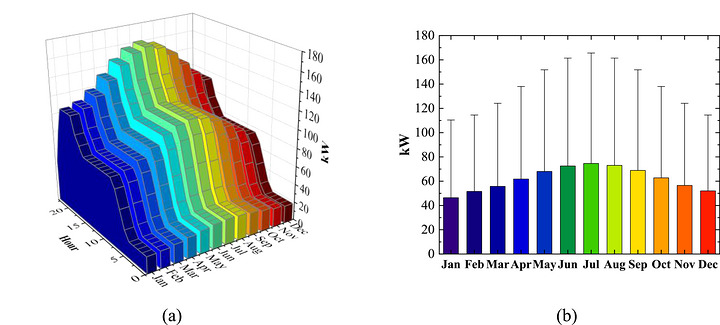
Electrical load profile: (a) Hourly–monthly (kW). (b) Monthly average load with minimum–maximum hourly variation.

Figure 2 shows the temporal variation of electrical load demand at both hourly and monthly scales. Figure 2a illustrates the 3D hourly–monthly electrical load profile derived from the given dataset. The x‐axis represents the hour of the day, the y‐axis denotes the months from January to December, and the z‐axis shows the load demand in kW. A consistent diurnal pattern is observed for all months, with minimum demand during late‐night and early‐morning hours and a sharp rise after 06:00. Peak demand occurs between 12:00 and 18:00. Seasonal variation is clearly visible, with summer months (June–August) exhibiting significantly higher peak loads, exceeding 160 kW, compared to winter months (December–February), where peak values remain below approximately 120 kW. This indicates increased energy usage during warmer periods. Figure 2b presents the monthly average load demand, with error bars indicating the minimum and maximum hourly loads for each month. The average demand increases gradually from January to July, reaching a maximum in mid‐summer, and then decreases toward December. Larger error bars during summer months reflect greater intra‐day variability, while smaller ranges in winter indicate more stable demand. Together, the figures highlight the combined effects of daily and seasonal variations on load behavior.

### 2.3 Resources

Site‐specific renewable resource variables, such as solar radiation, clearness index, temperature, and wind speed, are critical for realistic modeling in HOMER. NASA's Surface Meteorology and Solar Energy (SSE) database provided solar irradiation data for the research site [49]. HOMER Pro leverages long‐term averaged climate information from NASA, generally lasting 22 years (1983–2005), to offer trustworthy averages of temperature, wind speed, and solar radiation for the specified site [50].

#### 2.3.1 Solar Irradiance and Clearness Index

**FIGURE 3:**
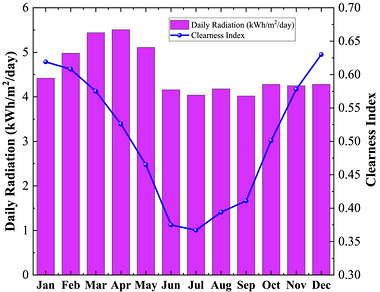
Solar GHI and clearness index for the site.

The average global horizontal irradiance (GHI) and clearness index at the research site are shown to vary monthly in Figure 3. The highest daily radiation is recorded in April (5.51 kWh/m^2^/day) and March (5.44 kWh/m^2^/day), while the lowest is recorded in July (4.04 kWh/m^2^/day). The clearness index has an inverse trend, with a minimum of 0.367 in July and a maximum of 0.630 in December. Although seasonal monsoon cloud cover considerably lowers sunlight from June to September, the yearly mean radiation of 4.56 kWh/m^2^/day indicates moderate solar potential. Increased solar radiation increases PV systems' capacity to produce electricity, while the clearness index, which measures air transparency, influences their efficiency by figuring out how much sunlight really reaches the panels [49].

#### 2.3.2 Wind Speed

**FIGURE 4:**
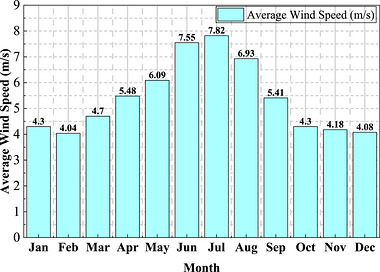
Monthly average wind speed for the site.

Figure 4 presents the monthly average wind speed distribution at 10 m above sea level. Wind speeds peak during July and June, reflecting strong monsoonal influence, while the lowest occurs in December and February. Moderate values are observed during spring and autumn, with May and September showing transitional patterns. The annual mean wind speed is 5.41 m/s, indicating favorable wind energy potential, particularly during the summer monsoon period, making this location suitable for hybrid renewable energy integration. Higher wind speeds enhance heat dissipation from solar panels, thereby improving their operating efficiency [51]. Conversely, cloud cover reduces solar irradiance and diminishes PV generation, though wind can help disperse clouds and support solar output. In addition, the performance of WTs is directly dependent on wind velocity; greater wind speeds lead to higher electricity production, while lower speeds result in reduced output [52].

#### 2.3.3 Temperature

**FIGURE 5:**
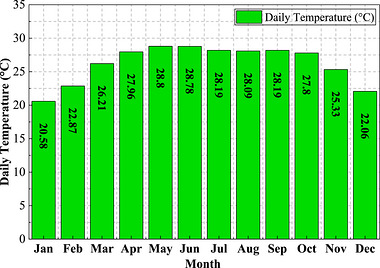
Monthly average temperature for the site.

Figure 5 Illustrates the Daily Temperature Profile of the Site, Showing an Average of 26.24°C. The Performance of Solar PV Systems Is Closely Influenced by Temperature Fluctuations, as Higher Temperatures Reduce Module Efficiency by Increasing the Internal Resistance of PV Panels [53].

### 2.4 Solar PV

The real power (*P_PV_*) of the PV panel under real operation and climatic conditions can be written in Equation (1) as [54]:

(1) $$P_{PV}=P_{STC}\;\left[1+\beta_{p}\left(T_{cell}-T_{STC}\right)\right]\frac{H_{t}}{H_{STC}}$$

where β_*p*_ is the power temperature coefficient, *P_STC_* is the power output at Standard Test Conditions (STC), *T_STC_* is the cell's surface temperature at STC and *T_cell_* is the actual cell surface temperature, *H_t_* is the real solar irradiance, and *H_STC_* is the irradiance at STC. The cell surface temperature *T_cell_* can be written in Equation (2) as [55]:

(2) $$T_{cell}=T_{\infty }+7.8\times 10^{-2}H_{t}$$

where *T*_∞_ is the ambient temperature.

### 2.5 Wind Turbine

Equation (3) enables the estimation of wind power at different heights as described in [56]. With the known turbine density, it is possible to determine wind acceleration variation, or conversely, calculate the density when wind acceleration is available. The fundamental power equation for WTs is expressed as:

(3) $$P=\frac{1}{2}\;C_{p}A\rho v^{3}$$

wherein, *P* is the WT power output in watts; ρ is the wind power density in W/m^2^; *v* is the wind velocity in m/s; *C_p_* is the rotor efficiency, and *A* is the rotor swept area in m^2^.

Moreover, wind energy modeling can be expressed in Equation (4) as [57]:

(4) $$E_{W}=\left\{\begin{array}{c} \begin{array}{c} \;\\ P_{rat}\left(\frac{V_{Z,t}-V_{cut-in}}{V_{rat}-V_{cut-in}}\right)\;V_{cut-in}<V_{Z,t}<V_{cut-off}\\ \; \end{array}\\ 0\;V_{Z,t}\leq \;V_{cut-in\;}OR\;V_{Z,t}\geq V_{cut-off} \end{array}\right.\;$$

where: *P_rat_* is the rated power of the WT at the rated wind speed *V_rat_*, *V*_*cut* − *in*_ and *V*_*cut* − *off*_ are the cut‐in and cut‐off wind speeds, and *V*_*Z*,*t*_ is the wind speed at the WT hub height (*h_Z_*) and it is calculated from Equation (5) [58]:

(5) $$V_{Z,t}=V_{0,t}\;{\left(\frac{h_{Z}}{h_{0}}\right)}^{\propto }$$

where, *V*_0,*t*_ is the wind speed at a certain elevation (*h*_0_) and ∝ is the wind shear coefficient.

### 2.6 Inverter

Because it transforms the DC power produced by solar panels into AC electricity for use inside the microgrid, the solar PV inverter is an essential part of microgrid systems. It also serves as a rectifier, converting AC to DC to enable BESS charging and promote efficient energy management [59]. Additionally, the inverter controls frequency and voltage, allowing solar energy to be seamlessly integrated into the grid while maximizing distribution. Equation (6) presents the simplified governing equation for this process as follows:

(6) $$P_{AC}=P_{DC}\;\eta_{Inverter}$$

In this case, *P_AC_* represents the AC output power in watts, *P_DC_* represents the DC input power in watts, and η_*Inverter*_ represents the inverter's efficiency. Observe that the efficiency of the inverter η_*Inverter*_ can vary depending on the power levels as well as the actual inverter technology being used and the operating conditions present.

### 2.7 BESS

State of Charge (SOC) of a BESS describes the proportion of available energy in the battery compared to its entire capacity, and it indicates the present energy level and is an indicator of the battery charge status. Equation (7) represents the model equation for the SOC of BESS in HOMER Pro:

(7) $$SOC\;\left(t\right)=SOC\left(T-1\right)+\frac{P_{charge}\;\left(t\right)\times \eta_{battery}-P_{discharge\;\left(t\right)}}{C_{battery}}$$

Here, *SOC*(*t*) represents the battery's state of charge at time t, *P_charge_* (*t*) represents the power used to charge the battery (kW), *P*_*discharge* (*t*)_ represents the power discharged from the battery (kW), η_*battery*_ is the battery efficiency, and *C_battery_* represents the battery's capacity (kWh).

### 2.8 Electrolyzer

Green hydrogen can be produced in a carbon‐free manner using water electrolysis with the help of an electrolyzer. This device utilizes excess renewable electricity from PV or WT systems to generate hydrogen, as expressed in Equation (8) [60]:

(8) $$H_{2}0\to H_{2}+\frac{1}{2}\;O_{2}$$

### 2.9 Hydrogen Tank

The hydrogen produced from the electrolyzer is required to be stored in a hydrogen tank, which can supply the hydrogen to an FC as required. The energy held by the HT can be derived in Equation (9) [61] below:

(9) $$E_{HT}=V_{HT}\;\rho_{H_{2}}LHV_{H_{2}}$$

where *V_HT_* refers to the volume of the tank and $\rho_{H_{2}}$ and $LHV_{H_{2}}$ refers to the hydrogen density and the lower heating value of the hydrogen, respectively.

### 2.10 Fuel Cell

Hydrogen stored in tanks can be converted back into electricity through a fuel cell, offering a carbon‐free method of power generation. The FC operates by combining hydrogen with oxygen from the air, producing electricity while releasing only water as a by‐product [62]. The efficiency of the FC can be determined using Equation (10) [63]:

(10) $$\eta_{FC}=\frac{{\bar{V}}_{c}\times n_{c,FC}\times I_{FC}}{Q\times HHV_{hyd}}\;$$

where ${\bar{V}}_{c}$ is the average voltage of a cell,*n*_*c*,*FC*_ is the total cell number of FC, *Q* is the molar flow rate of hydrogen (mol/s), and *I_FC_* is the current in FC. *HHV_hyd_* represents the gross calorific value of hydrogen.

### 2.11 HOMER Pro

**FIGURE 6:**
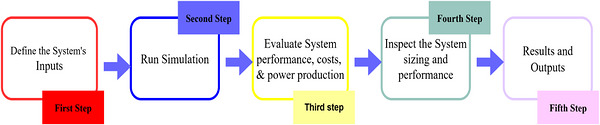
Architecture of HOMER Pro software [16].

HOMER (Hybrid Optimization of Multiple Energy Resources) Pro is the industry standard for microgrid design and optimization, building on decades of knowledge in distributed power systems that incorporate fossil fuel production, storage, and renewable energy sources [64]. HOMER Pro, created by the National Renewable Energy Laboratory (NREL) in the United States, simulates system performance and lifetime costs, including capital and operating expenses, to aid in the design of microgrids powered by renewable energy [65]. By offering modeling, optimization, and sensitivity analysis while taking factors like load growth and future fuel costs into account, the program helps overcome the difficulties in microgrid design for distant places [66]. By estimating annualized costs with an emphasis on component‐specific charges, HOMER Pro affects NPC and LCOE [67]. By entering factors such as component costs, load demand, data on renewable resources, and technical requirements, users may run simulations [68]. Figure 6 gives the general architecture, which aids in the selection of the optimal technical and financial options [69]. The modular structure of HOMER Pro comprises elements for data input, simulation, optimization, and outcomes analysis. By combining renewable energy resources with different system components, it is possible to optimize distributed energy systems and microgrids. In order to achieve the most efficient system design, it also determines the optimal system configuration in terms of LCOE and NPC by assessing the availability of renewable energy sources (such as solar and wind), electric demand, and component performance.

The model equations for NPC and LCOE or COE in HOMER pro are represented as follows in Equations (11) and (12), respectively.

(11) $$\mathrm{NPC}=C_{\mathrm{cap}}+\sum_{t=1}^{T}\frac{C_{\mathrm{om}}\left(t\right)+C_{\mathrm{fuel}}\left(t\right)}{{\left(1+r\right)}^{t}}$$

where *C*_cap _is the capital cost of the system, *C*_om_(*t*) is the operation and maintenance cost in year t, *C*_fuel_(*t*) is the fuel cost in year t (biomass/diesel fuel), *r* is the discount rate, and *T* is the project lifetime (years).

(12) $$\mathrm{LCOE}=\frac{\sum_{t=1}^{T}\left(C_{\mathrm{cap}}+C_{\mathrm{om}}\left(t\right)+C_{\mathrm{fuel}}\left(t\right)\right)}{\sum_{t=1}^{T}E_{\mathrm{total}}\left(t\right)}$$

where *LCOE* is the levelized cost of energy (in $/kWh), *C*_cap_ is the total capital cost of the system (initial investment), *C*_om_(*t*) is the operating and maintenance costs in year t, *C*_fuel _(*t*) is fuel costs in year t (for biomass, diesel, or other fuels), *E*_total_(*t*) is the total electricity generated in year *t* (in kWh), *T* is the lifetime of the system in years.

Moreover, the *LCOE* and payback time in monetary terms (*PBTM*) can be calculated using the cost of environmental damage caused by carbon dioxide *C*_CO2_, as shown in the following Equations (13) and (14) [58]:

(13) $$LCOE=\frac{\left(\frac{r{\left(1+r\right)}^{n}}{{\left(1+r\right)}^{n}-1}\right)\times C+C_{O\&M}-C_{\mathrm{CO}2}}{E_{t}}$$

(14) $$PBTM=\frac{C}{\frac{{\left(1+r\right)}^{n}-1}{r{\left(1+r\right)}^{n}}\left(C_{CO2}-C_{O\&M}\right)}$$

where: *C* is the capital cost of the system in $, $C_{O\&M}$ denotes the cost of operation and maintenance ($/year), *E*_t_ is the annual energy produced by the system (kWh/year), *n* is the device lifetime (25 years), and *r* is the annual inflation rate.

The following Equation (15) calculates the LCOH in HOMER Pro.

(15) $$LCOH=\frac{C_{ann,\;tot\;}-v_{elec\;}\left(E_{prim,AC}+E_{prim,DC}+E_{def\;}+E_{grid,\;sales\;}\right)}{M_{H_{2}}}\;$$

where *C*_ann, tot _, is the total annualized cost, *v*_elec _ is the value of electricity, *E_prim_* is the primary electrical load, E_def _ is the deferrable load, E_grid, sales _, is the total energy sold to the grid, and $M_{H_{2}}$ is the total hydrogen production.

The cost of environmental damage ($C_{CO_{2}}$) caused by *CO*_2_ gas can be calculated by the following equation (16) [70].

(16) $$C_{CO_{2}}=EF_{CO_{2}}\;\times E_{t}\times \emptyset_{CO_{2}\;}$$

where: $EF_{CO_{2}}$ represents the *CO*_2_ emission factor of the electric power generation system (kg CO_2_/kWh) [71], $\emptyset_{CO_{2}\;}$ represents the carbon social cost ($/ton CO_2_), which may be considered as $70/ton CO_2_ [72].

**FIGURE 7:**
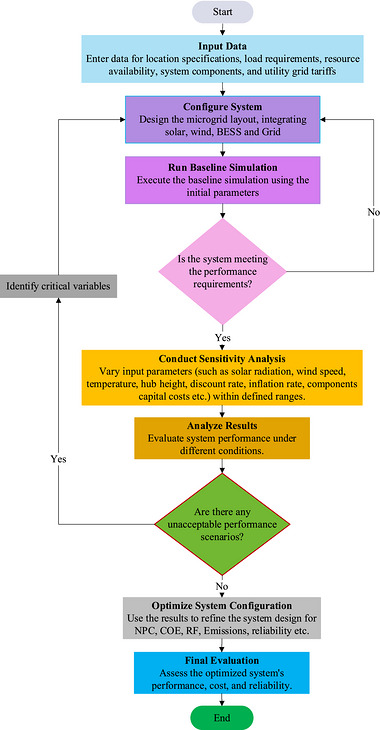
Methodology flowchart for the proposed work.

Figure 7 illustrates a structured methodology flowchart for microgrid design and optimization. Data collection and system configuration are the first steps in the process, which is then followed by a baseline simulation to verify performance standards. The pipeline moves on to sensitivity analysis, load dispatch options, and overall performance evaluation if the outcomes satisfy the criteria. Until optimization is attained, all problems found are redirected for more improvement. In order to guarantee an effective microgrid design, the system assesses cost, dependability, and environmental effects at the last step [73].

**FIGURE 8:**
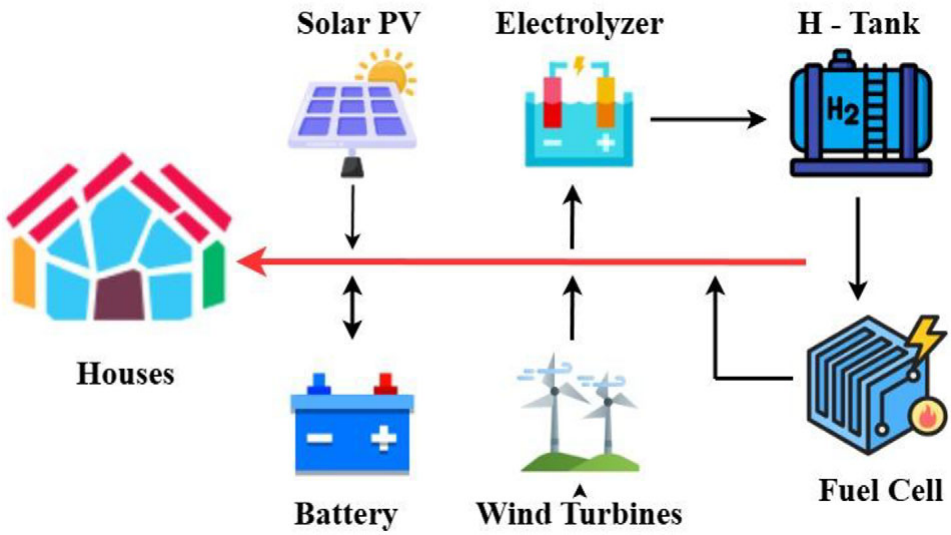
HRES schematic diagram of the proposed microgrid.

Figure 8 presents the proposed off‐grid HRES designed to meet the electricity needs of rural households. The system combines solar PV and WTs as the main renewable sources, while batteries are included to provide short‐term energy storage and ensure a smooth supply during daily fluctuations. When electricity generation exceeds demand, the surplus energy is directed to electrolyzers, which produce hydrogen that is stored in an H_2_ tank for long‐term use. During periods of low renewable generation, FCs convert the stored hydrogen back into electricity, functioning as a backup supply. This integrated architecture ensures continuous, reliable, and low‐carbon energy delivery, offering a sustainable approach to address rural electrification challenges in remote coastal regions.

**FIGURE 9:**
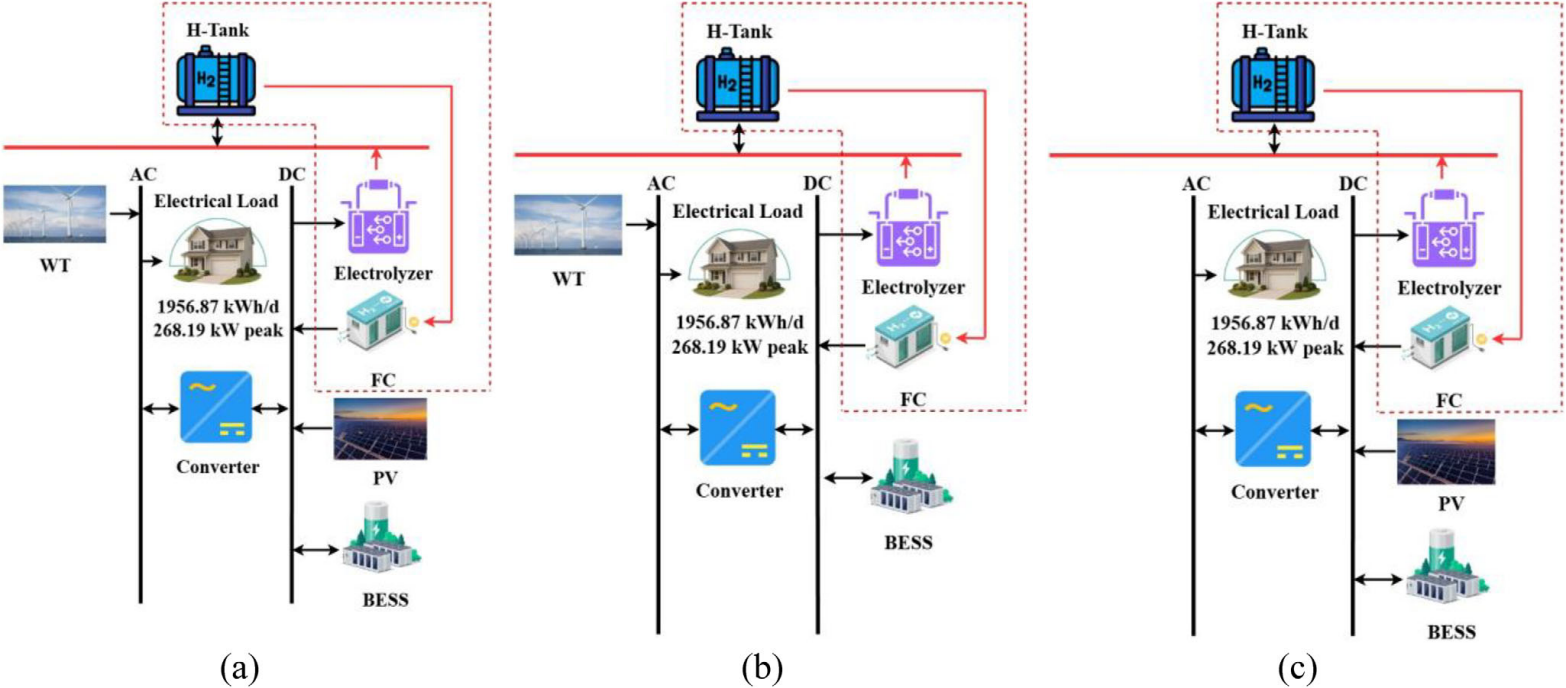
HOMER Pro–based schematic representations of three off‐grid hybrid renewable microgrid configurations: (a) Case A, (b) Case B, and (c) Case C.

Figure 9 illustrates three alternative off‐grid HRES configurations evaluated using HOMER Pro to supply electricity to a rural community with a daily demand of 1956.87 kWh and a peak load of 268.19 kW. Each configuration integrates renewable generation, energy storage, and power conversion components, but differs in the primary energy sources employed. Figure 9a presents Case A, which combines PV and WTs with a BESS and a hydrogen subsystem consisting of an electrolyzer, a hydrogen storage tank, and FC. AC and DC buses are interconnected through a bidirectional converter, enabling flexible power exchange. Excess renewable electricity is first used to charge the BESS and then to produce green hydrogen via electrolysis for long‐term storage. During periods of low renewable generation, the FC converts stored hydrogen back into electricity, ensuring high reliability. The complementary nature of solar and wind resources, together with dual storage (battery and hydrogen), results in the lowest net present cost and cost of energy, making Case A the best‐performing scenario. Figure 9b shows Case B, which relies primarily on wind energy supported by BESS and hydrogen storage. The absence of PV reduces resource diversity, leading to higher costs and lower system flexibility. Figure 9c illustrates Case C, a PV‐dominant system with BESS and hydrogen storage, which suffers from higher capital costs and stronger solar intermittency. Overall, the comparison highlights the techno‐economic advantage of integrating both PV and wind with hybrid storage, as demonstrated in Case A.

The specifications of the primary microgrid components, including renewable energy sources, PV modules, WT, inverters, FCs, electrolyzers, H_2_ storage, and BESS, are summarized in Table 2. Component selection was carried out in accordance with HOMER Pro criteria to ensure system dependability, cost‐effectiveness, and long‐term sustainability. Focus was on efficiency, durability, and integration, strengthening the microgrid's capacity to meet community energy needs while promoting resilience and sustainability.

**TABLE 2:** Technical specifications of the components.

Parameters	PV	WT	Inverter	Electrolyzer	FC	BESS
Rated capacity	0.345 kW	3 kW	—	400 KW	140 kW	1890 Ah
Efficiency	17.8%	13%	95%	85%	—	80%
Hub Height	—	17 m	—	—	—	—
Life Time	25yr	20yr	15yr	15yr	50,000hr	12yr

Table 3 outlines the per‐unit costs of PV, WT, Inverter, Electrolyzer, FC, and BESS, including capital, replacement, and O&M costs for economic feasibility evaluation with current references. This integrated methodology, incorporating detailed simulations of various energy generation and storage systems, provides a solid basis for the subsequent analysis and discussion focused on optimizing energy efficiency and enhancing sustainability.

**TABLE 3:** Per unit cost of components.

Parameters	PV	WT	Inverter	H_2_ Tank	Electrolyzer	FC	BESS
Capital cost	$280.00	$862.50	$280.00	$1.50	$100.00	$450.00	$1,259
Replacement cost	$280.00	$862.50	$280.00	$0.50	$100.00	$400.00	$1,100
O & M cost	$8.00	$0.0	$0.0	$0.60	$8.00	$0.15	$5
Reference	[84]	[85]	[86]	[21]	[21]	[21]	[87]

## 3 Results and Discussion

In the simulation study, a total of 2,396 possible microgrid solutions were investigated. Out of these, 930 configurations were found to be feasible, meeting all operational and technical constraints. However, 1,466 configurations were deemed infeasible due to capacity shortage limitations, meaning they could not adequately meet the required load demand. Additionally, 388 configurations were omitted from the analysis — none due to infeasibility, but 372 were excluded for lacking a converter component. These results highlight the importance of appropriate component selection, particularly converters, and capacity optimization to ensure a reliable and technically viable HRES design for the proposed microgrid. Using hourly time‐series analysis over a 25‐year horizon, three scenarios integrating PV, WT, FCs, electrolyzers, H_2_ storage, and BESS were evaluated. As summarized in Table 4.

**TABLE 4:** Summary of different scenarios.

Components	Configuration
PV‐WT‐BESS‐Converter‐Electrolyzer‐FC‐H_2_ Tank	Case‐A
WT‐BESS‐Converter‐Electrolyzer‐FC‐H_2_ Tank	Case‐B
PV‐BESS‐Converter‐Electrolyzer‐FC‐H_2_ Tank	Case‐C

### 3.1 Techno‐Economic Assessment of the Microgrid

**FIGURE 10:**
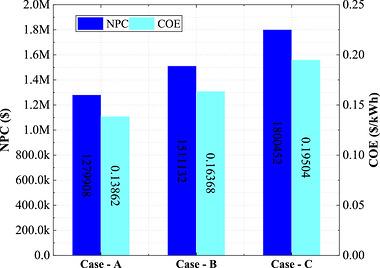
Techno‐economic comparison across scenarios: NPC and COE.

Figure 10 presents the techno‐economic comparison of three system configurations (Cases A, B, and C) based on the NPC and COE. Among the scenarios, Case A demonstrates the most cost‐effective performance, with an NPC of $1 279 908 and a COE of $0.13862/kWh, making it the optimal configuration. In contrast, Case B exhibits a higher NPC of $1 511 132 and COE of $0.16368/kWh, while Case C records the highest NPC and COE values at $1 800 452 and $0.19504/kWh, respectively. The analysis clearly indicates that incorporating optimized renewable and storage components, as in Case A, results in the lowest lifecycle cost and energy price, ensuring both economic and operational sustainability for rural microgrid applications.

**FIGURE 11:**
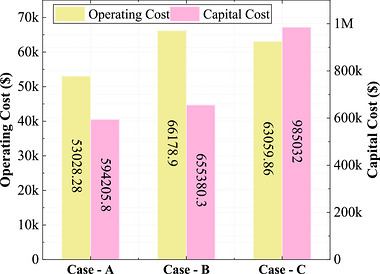
Techno‐economic comparison across scenarios: Operating and Capital Costs.

Figure 11 compares the techno‐economic performance of three microgrid configurations in terms of capital and operating costs. Among the scenarios, Case A exhibits the lowest overall expenditure, with a capital cost of $594 205.80 and an operating cost of $53 028.28, making it the most cost‐effective option. Case B records slightly higher values, with a capital cost of $655 380.30 and an operating cost of $66 178.90. Conversely, Case C presents the highest costs, showing a capital investment of $985 032 and an operating cost of $63 059.86. The analysis confirms that Case A offers the most economically balanced configuration, minimizing both initial investment and recurring expenses while maintaining system efficiency and long‐term sustainability for rural electrification.

### 3.2 Optimal Scenario

**FIGURE 12:**
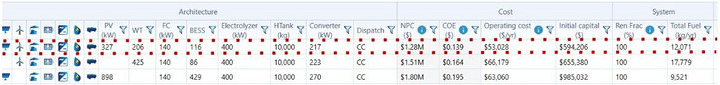
HOMER Pro simulation results indicating optimal microgrid configuration.

From the preceding analysis, Case‐A is identified as the most suitable option for microgrid planning based on its superior economic and environmental performance. It offers a well‐balanced solution characterized by the lowest NPC and COE, along with moderate capital and operating expenses. Additionally, Case‐A demonstrates the highest RF, minimal greenhouse gas emissions, and acceptable levels of excess electricity and unmet load, while ensuring efficient grid transactions. Figure 12 illustrates the HOMER Pro simulation outcomes, highlighting the optimal configuration of the proposed hybrid microgrid system.

**FIGURE 13:**
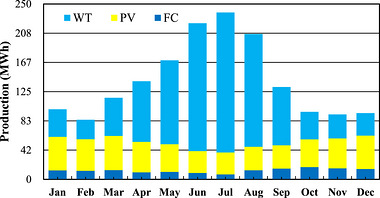
Monthly energy production for case A.

Case A represents the most cost‐effective and efficient microgrid configuration, achieving the lowest NPC, COE, capital, and operating expenses. As shown in Figure 13, WT dominates energy generation, contributing about 70–85% annually. PV systems account for 15–25%, particularly compensating during low‐wind months, while FCs contribute 5–8% to maintain system stability. The coordinated operation of WT, PV, and FC ensures reliable, complementary seasonal performance, with respective annual energy shares of roughly 70%, 22%, and 8%, supporting sustainable and continuous off‐grid power generation. Although a conventional BESS model was employed in this study using HOMER Pro, the specific battery chemistry was not explicitly defined and is assumed to represent typical lithium‐ion characteristics. In practical implementations, battery performance is strongly influenced by environmental conditions, particularly in coastal regions characterized by high temperature, humidity, and frequent charge–discharge cycles. Advanced battery technologies, such as lithium iron phosphate (LiFePO_4_), solid‐state batteries, and systems equipped with enhanced thermal management, offer improved cycle life, higher thermal stability, and greater resilience under harsh operating conditions. The integration of such next‐generation storage solutions could potentially reduce degradation rates, lower replacement costs, and ultimately decrease the COE. Therefore, future work should focus on comparative techno‐economic analysis of different battery chemistries and the incorporation of advanced thermal management strategies to further optimize system performance and long‐term sustainability. In the current system design, hydrogen produced by the electrolyzer is primarily utilized within the microgrid through a power‐to‐gas‐to‐power pathway, where it is stored and later converted back into electricity using a fuel cell. However, expanding the utilization of the hydrogen subsystem beyond internal power generation offers significant opportunities to enhance overall system efficiency and economic performance. In coastal and rural regions such as Char Ishwar, surplus green hydrogen could be exported and utilized as a clean fuel for local agricultural machinery, fishing boats, and other hard‐to‐decarbonize applications. Such cross‐sectoral integration would enable multi‐energy complementarity, improve hydrogen infrastructure utilization rates, and create additional revenue streams that could further reduce the COE. Therefore, future research should investigate the techno‐economic feasibility of hydrogen export pathways, sector coupling strategies, and integration with emerging regional hydrogen markets to maximize the value of green hydrogen in decentralized energy systems.

**FIGURE 14:**
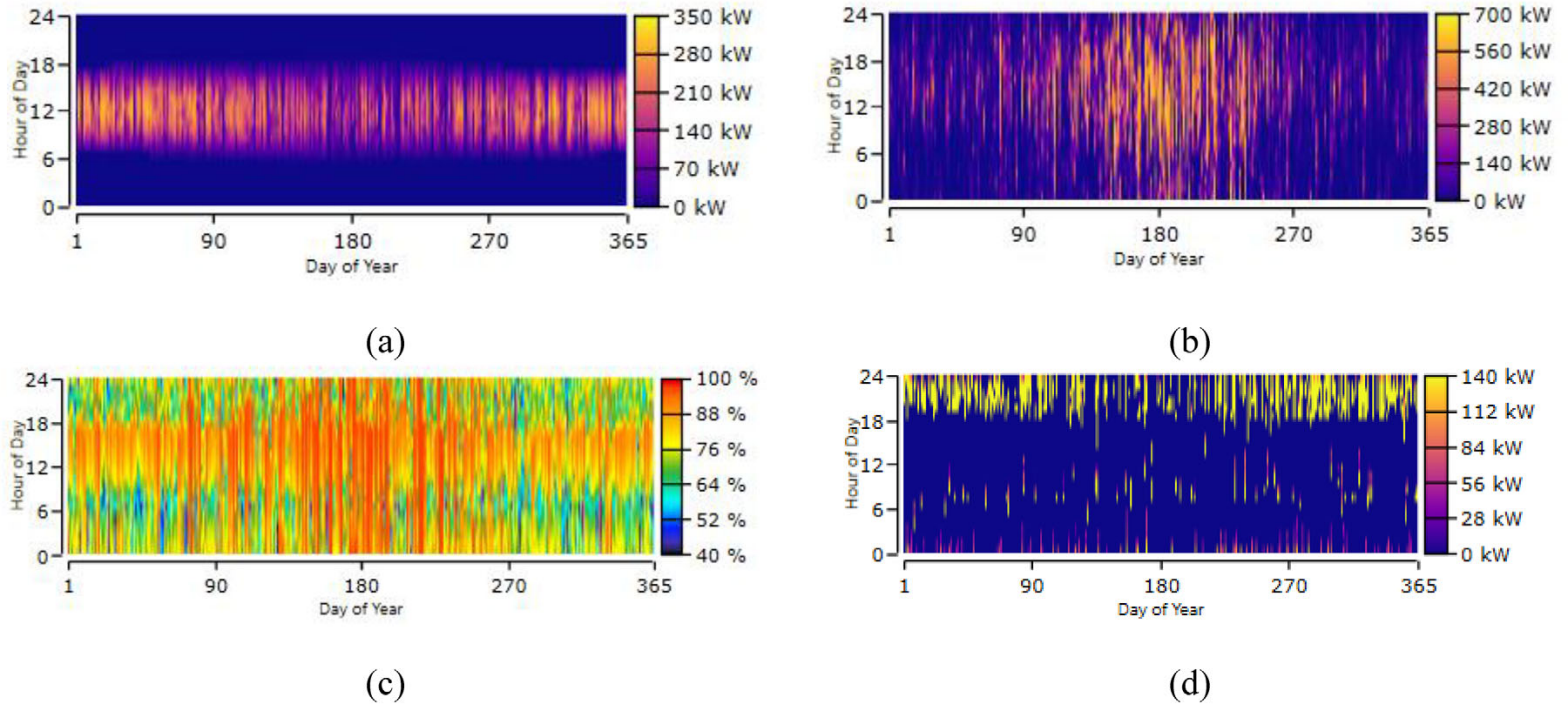
Performance of the main components of the proposed hybrid renewable microgrid: (a) PV power output, (b) WT power output, (c) SOC of BESS, (d) FC power output.

Figure 14 presents a comprehensive performance overview of the proposed hybrid renewable microgrid, highlighting the individual and collective roles of its key components in delivering reliable off‐grid power. Figure 14a illustrates the hourly and seasonal performance of the 327 kW Trina Solar PV system, which produces 483,513 kWh annually with a specific yield of 1,477 kWh/kW and a PV penetration of 67.7%. Peak output of up to 350 kW occurs between 09:00 and 15:00, with higher generation in summer and lower output in winter. Due to low capital and O&M costs, the system achieves a low COE of $0.0201/kWh. Figure 14b shows the 618 kW wind turbine system producing 1,058,688 kWh/year over 7,222 hours, with significant variability and frequent outputs between 175 and 525 kW. Wind generation complements solar by operating during both day and night. Figure 14c depicts the 875 kWh BESS SOC profile, showing effective cycling between 40% and 100% and providing 6.44 hours of autonomy. Finally, Figure 14d highlights the 140 kW fuel cell, which supplies 150,883 kWh annually during renewable shortfalls, ensuring a continuous and reliable power supply. Together, these figures demonstrate the technical robustness and operational synergy of the proposed hybrid microgrid system.

**FIGURE 15:**
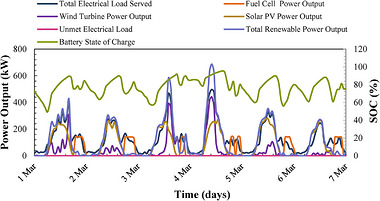
Renewable Energy Output and Load Demand Profile (March 1–7).

Figure 15 illustrates the renewable energy output and electrical load profile from March 1–7, highlighting the interaction among various system components. WTs provide the primary power contribution, frequently exceeding 500 kW and peaking near 700 kW, while solar PV generation reaches about 200 kW during daylight hours. FCs supplement the supply during low renewable output, delivering up to 150 kW. The total load served closely tracks demand, with unmet load remaining nearly zero, indicating stable operation. SOC fluctuates between 60% and 100%, reflecting efficient charging during surplus generation and discharge during deficits. Overall, the system demonstrates effective energy balancing and reliable supply through coordinated operation of wind, solar, FCs, and battery storage.

**FIGURE 16:**
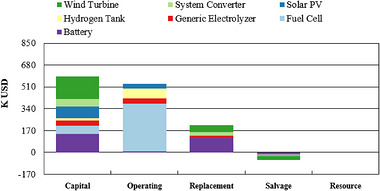
Cost breakdown for system components.

Figure 16 illustrates the detailed cost breakdown for each component of the HRES, segmented into capital, operating, replacement, salvage, and resource costs. The total system cost amounts to approximately $1.28 million, with capital $594,206 and operating $533,443 representing the largest financial burdens. WTs and FCs dominate the capital and operating expenditures, respectively, with FCs alone contributing around $431,906 due to high maintenance and operational demands. Replacement costs total approximately $211,972, primarily from batteries $112,513 and system converters $25,808. Negative salvage values of about –$59,713, mainly from WTs (–$31,943) and batteries (–$15,514), indicate decommissioning expenses at the end of system life. Hydrogen tanks ($92,585) and electrolyzers ($95,160) contribute modestly to total costs. Overall, the figure highlights that operational and maintenance activities constitute a substantial portion of lifecycle costs, underscoring the importance of durability and efficiency optimization in system design.

**FIGURE 17:**
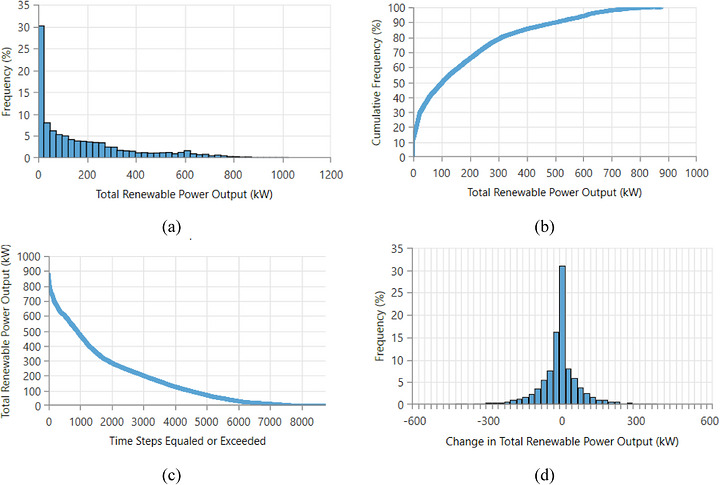
Distribution, cumulative behavior, duration characteristics, and hourly variability of total renewable power output: (a) histogram, (b) CDF, (c) duration curve, and (d) histogram of hourly output changes.

Figure 17 presents a comprehensive statistical characterization of total renewable power output using distributional and duration‐based analysis. Figure 17a shows the histogram of total renewable power output, indicating a highly right‐skewed distribution. Approximately 30% of observations fall below 50 kW, highlighting frequent low‐generation periods, while outputs above 600 kW occur infrequently (<5%), with a maximum approaching 900 kW. This reflects the intermittency inherent in renewable resources. Figure 17b illustrates the cumulative distribution function (CDF). Nearly 50% of the time, total renewable output remains below 120 kW, about 80% of values are below 300 kW, and 95% are under 600 kW. This confirms that high power outputs are relatively rare and concentrated in limited periods. Figure 17c shows the renewable power duration curve, with peak output of approximately 900 kW occurring for a small fraction of the year and outputs below 200 kW prevailing for more than half of the ∼8,000 h period, indicating limited high‐output availability. Figure 17d illustrates hourly variability, with most power changes concentrated within ±100 kW, a strong central peak near 0 kW, and infrequent extreme ramps reaching ±400 kW. Overall, these figures demonstrate significant variability in renewable generation, emphasizing the necessity of energy storage and complementary generation for reliable microgrid operation.

**FIGURE 18:**
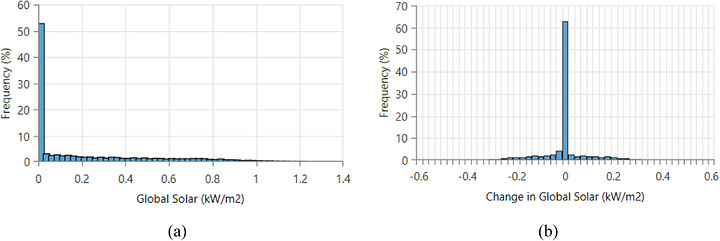
Distribution and short‐term variability of global solar irradiance: (a) histogram of irradiance levels and (b) histogram of hourly irradiance changes, showing dominant low values and mostly small fluctuations around zero.

Figure 18 illustrates the statistical characteristics and short‐term variability of global solar irradiance. The histogram in Figure 18a shows a highly right‐skewed distribution, with approximately 52% of observations concentrated near zero irradiance, corresponding to nighttime and low‐sun conditions. Most nonzero values lie below 0.4 kW/m^2^, while peak irradiance rarely exceeds 1.0–1.2 kW/m^2^, indicating limited periods of strong solar availability. Figure 18b presents the frequency distribution of hourly changes in global solar irradiance. A pronounced peak at 0 kW/m^2^ accounts for nearly 63% of occurrences, reflecting minimal hour‐to‐hour variation during stable daylight or nighttime conditions. Moderate fluctuations within ±0.2 kW/m^2^ dominate the remaining observations, whereas extreme ramps beyond ±0.4 kW/m^2^ are rare (<2%). Overall, the figures demonstrate strong intermittency but relatively smooth short‐term dynamics, underscoring the importance of temporal smoothing and storage integration in solar‐dominated energy systems.

### 3.3 Sensitivity Analysis Results

Sensitivity analysis in HOMER Pro was conducted to examine how variations in key input parameters influence the performance of energy system models and to assess the robustness of microgrid configurations under different operating conditions. As shown in Table 5, critical variables including solar irradiance, wind speed, ambient temperature, turbine hub height, nominal discount rate, and inflation rate were varied by ±20% to capture realistic environmental and economic uncertainties. This approach allows the identification of parameters that have the greatest impact on system cost and operational performance. Consequently, the analysis supports informed decision‐making by guiding optimal system design choices. The present analysis utilizes long‐term averaged meteorological data, which may not fully capture the impact of extreme weather events such as cyclones, storm surges, and prolonged periods of low solar and wind availability that are characteristic of coastal regions like Char Ishwar. In this context, system resilience is enhanced through the integration of hybrid renewable sources and dual energy storage mechanisms. While the BESS provides short‐term balancing and daily load support, the hydrogen subsystem, comprising the electrolyzer, hydrogen tank, and fuel cell, offers long‐duration energy storage capable of sustaining the system during extended renewable deficits. This power‐to‐gas‐to‐power pathway enables energy shifting across longer timescales, thereby improving resilience against temporary resource unavailability. However, under extreme “black swan” scenarios involving multi‐day or seasonal disruptions, additional design considerations such as increased hydrogen storage capacity, reserve margins, or auxiliary backup systems may be required. Therefore, future work should incorporate extreme‐event scenario modeling and resilience‐oriented optimization to ensure robust microgrid operation under severe climatic conditions. The findings also facilitate adaptive microgrid planning, ensuring reliable operation under potential fluctuations in climate conditions and financial assumptions.

**TABLE 5:** List of input sensitive variables with values.

Input sensitive variable	values
Solar radiation (kWh/m^2^/day)	2.74, 3.65, **4.56**, 5.46, 6.38
Wind speed (m/s)	3.24, 4.32, **5.41**, 6.48, 7.56
Temperature (°C)	15.74, 20.1, **26.23**, 31.48, 36.73
Hub‐height (m)	10.2, 13.6, **17**, 20.4, 23.8
Electric Load(kWh/d)	1174.12, 1565.5, **1956.87**, 2348.24, 2739.61

*Note*: The bold values represent the middle or base values for sensitivity analysis.

**FIGURE 19:**
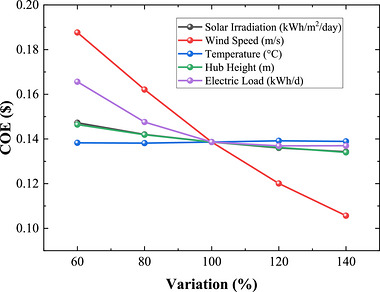
Impact of parameter variations on COE in microgrid sensitivity analysis.

Figure 19 illustrates the percentage‐based sensitivity of the COE to variations (60–140%) in key system parameters relative to the nominal 100% case (COE = $0.139/kWh). Wind speed shows the highest sensitivity: decreasing it to 60% raises the COE to about $0.188/kWh (+35%), while increasing it to 140% lowers the COE to approximately $0.106/kWh (−24%). Electric load is the second most influential factor, increasing COE by nearly 20% at 60% load and slightly reducing it (≈1%) at 140%. Solar irradiation and hub height have moderate impacts, each reducing COE by about 3–4% at 140%. Temperature variations cause minimal change (within ±0.5%), indicating low sensitivity.

Table 6 presents the sensitivity ranking of key parameters affecting the COE in the system. Parameters are ordered from the highest to the lowest influence based on the magnitude of COE variation under percentage changes. The table explains how each parameter impacts system economics, identifying wind speed and electric load as the most influential factors, while solar irradiation and hub height show moderate effects. Temperature exhibits minimal influence, indicating limited sensitivity of COE to thermal variations.

**TABLE 6:** Sensitivity ranking of parameters affecting COE with explanations.

Rank	Parameter	Reason
1st	Wind Speed	Exhibits the highest sensitivity to COE variations. A decrease to 60% increases COE by about 35%, while an increase to 140% reduces COE by nearly 24%, indicating strong dependence of system economics on wind resource availability.
2nd	Electric Load	Shows substantial influence on COE, with approximately a 20% increase at 60% load and marginal reduction at higher load levels, reflecting the impact of demand scaling on system utilization and cost recovery.
3rd	Solar Irradiation	Demonstrates moderate sensitivity, where higher irradiation levels lead to noticeable COE reductions (around 3–4%), highlighting the role of solar resource availability in lowering energy costs.
4th	Hub Height	Has a limited but observable effect on COE, with small reductions (about 3%) at higher variations, indicating marginal gains from increased wind capture efficiency.
5th	Temperature	Least sensitive parameter, with COE variations remaining within ±0.5%, suggesting minimal impact of temperature changes on overall system cost performance.

**FIGURE 20:**
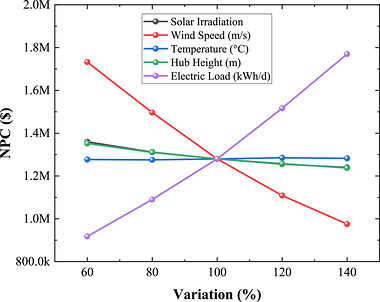
Impact of parameter variations on NPC in microgrid sensitivity analysis.

Figure 20 presents the sensitivity of the NPC to percentage variations from 60% to 140% of key system parameters relative to the baseline case at 100%, where the NPC is approximately $1.28 million. Wind speed has the most pronounced influence: reducing it to 60% increases the NPC to nearly $1.73 million, corresponding to about a 35% rise, while increasing it to 140% lowers the NPC to around $0.98 million, representing a reduction of approximately 23%. Electric load also shows strong sensitivity, with the NPC decreasing to about $0.92 million at 60% load and increasing sharply to almost $1.78 million at 140%. Solar irradiation and hub height have moderate effects, leading to NPC changes of roughly 3–4%, whereas temperature variations cause negligible changes, remaining within about ±1%.

Table 7 summarizes the sensitivity ranking of key system parameters based on their impact on the NPC. Parameters are ordered according to the magnitude of NPC variation resulting from percentage changes between 60% and 140% of their nominal values. It identifies electric load and wind speed as the dominant factors influencing overall system cost, while solar irradiation and hub height exhibit moderate effects. Temperature shows negligible influence, indicating that NPC is largely insensitive to thermal variations within the examined range.

**TABLE 7:** Sensitivity ranking of parameters affecting NPC with explanations.

Rank	Parameter	Reason
1st	Electric Load	Highest NPC sensitivity, ranging from about −28.19% at 60% to +38.53% at 140%, showing the strongest cost dependence on demand level.
2nd	Wind Speed	Strong inverse effect on NPC, increasing by about 35.37% at 60% and decreasing by about 23.77% at 140%, indicating a major influence of wind resource strength.
3rd	Solar Irradiation	Moderate impact; NPC varies within about +6.25% to −3.12%, reflecting secondary dependence on solar resource availability.
4th	Hub Height	Limited influence; NPC changes within about +5.63% to −3.33%, indicating marginal economic benefit from hub‐height adjustments.
5th	Temperature	Minimal effect; NPC remains within about ±0.41%, showing negligible sensitivity to temperature variation.

**FIGURE 21:**
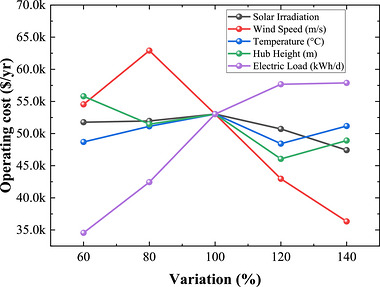
Impact of parameter variations on Operating Cost in microgrid sensitivity analysis.

Figure 21 illustrates the percentage‐based sensitivity of the annual operating cost to variations from 60% to 140% of key system parameters relative to the nominal case at 100%, where the operating cost is approximately $53 000 per year. Electric load exhibits the strongest positive sensitivity: reducing it to 60% lowers operating cost to about $34 500, corresponding to a decrease of nearly 35%, while increasing it to 140% raises the cost by about 9%. Wind speed shows strong inverse sensitivity, with operating cost increasing by about 19% at 80% wind speed and decreasing sharply by approximately 32% at 140%. Hub height and solar irradiation demonstrate moderate impacts, reducing operating cost by roughly 10–13% at higher levels. Temperature has a minor effect, with variations generally within 8%, indicating limited sensitivity.

Table 8 presents the sensitivity ranking of key parameters based on their influence on the annual operating cost of the system. Parameters are ordered according to the magnitude of cost variation resulting from percentage changes between 60% and 140% of their nominal values. It identifies electric load and wind speed as the most influential factors affecting operating expenses, while hub height and solar irradiation show moderate impacts. Temperature has the least effect, indicating limited sensitivity of operating cost to thermal variations within the analyzed range.

**TABLE 8:** Sensitivity ranking of parameters affecting Operating Cost with explanations.

Rank	Parameter	Reason
1st	Electric Load	Most influential parameter. Reducing load to 60% lowers operating cost by roughly one‐third, while increasing it to 140% raises cost by about 10%, showing strong dependence of annual operating expenses on demand level.
2nd	Wind Speed	Large inverse effect on cost. Higher wind speed (140%) reduces operating cost by about 30%, whereas lower speed (80–60%) increases it by up to nearly 20%, indicating major sensitivity to wind resource availability.
3rd	Hub Height	Moderate impact: variations between 60% and 140% cause operating cost changes of around 5–13%, reflecting the role of hub height in improving wind energy capture and reducing fuel usage.
4th	Solar Irradiation	Smaller but noticeable influence, with maximum deviations of about 10% from the baseline operating cost as irradiation changes, indicating secondary dependence on solar resource intensity.
5th	Temperature	Least sensitive parameter; operating cost changes remain within roughly ±10%, showing that temperature variations have a limited effect on system operation and fuel consumption.

**FIGURE 22:**
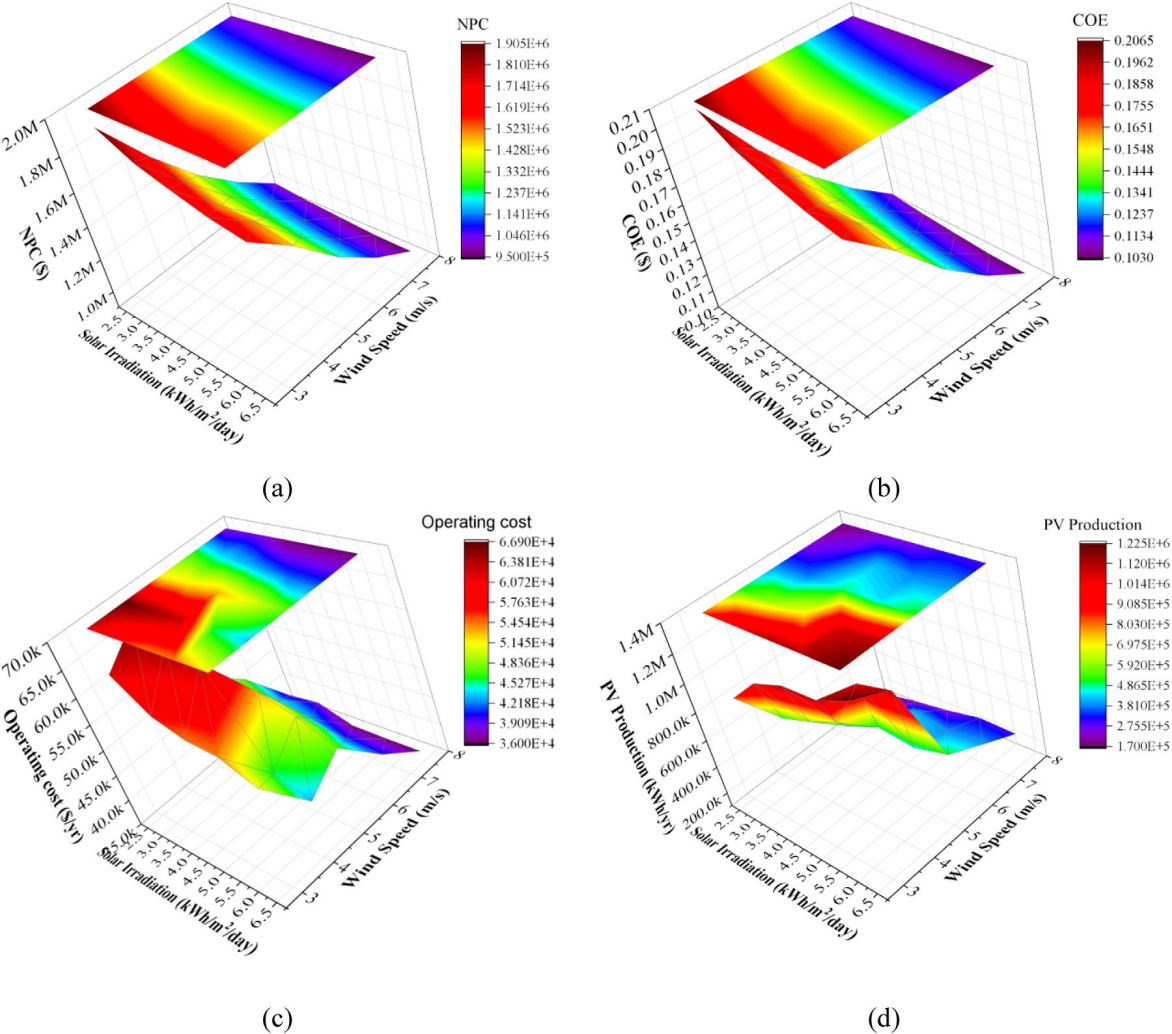
Sensitivity of solar irradiation and wind speed on system performance: (a) NPC, (b) COE, (c) Operating Cost, and (d) Annual PV production.

Figure 22 illustrates the sensitivity of system performance to variations in solar GHI and wind speed. Figure 22a shows that the NPC is strongly influenced by GHI, decreasing significantly as irradiation increases. At the base condition of 4.5775 kWh/m^2^/day and base wind speed 5.41 m/s, the NPC is approximately $1.30 million. Reducing GHI to about 2.75 kWh/m^2^/day results in an NPC increase of nearly 12%, while increasing GHI to around 6.4 kWh/m^2^/day reduces NPC by more than 35% due to higher renewable energy availability. Figure 22b indicates a similar trend for the COE, which declines from approximately 0.21$/kWh at low irradiation to about 0.13$/kWh at higher irradiation levels. Figure 22c demonstrates that higher GHI and wind speed jointly enhance the RF, increasing it from nearly 70% to above 95%, while simultaneously reducing operating costs. Figure 22d shows a substantial rise in annual renewable electricity generation, with total PV–wind production exceeding 1.1 GWh under favorable resource conditions. In contrast, variations in wind speed at fixed GHI exhibit comparatively moderate economic impacts, primarily improving energy yield and renewable penetration. Overall, the results confirm that GHI is the dominant factor affecting system economics, while wind speed provides complementary benefits by enhancing energy production and system reliability.

**FIGURE 23:**
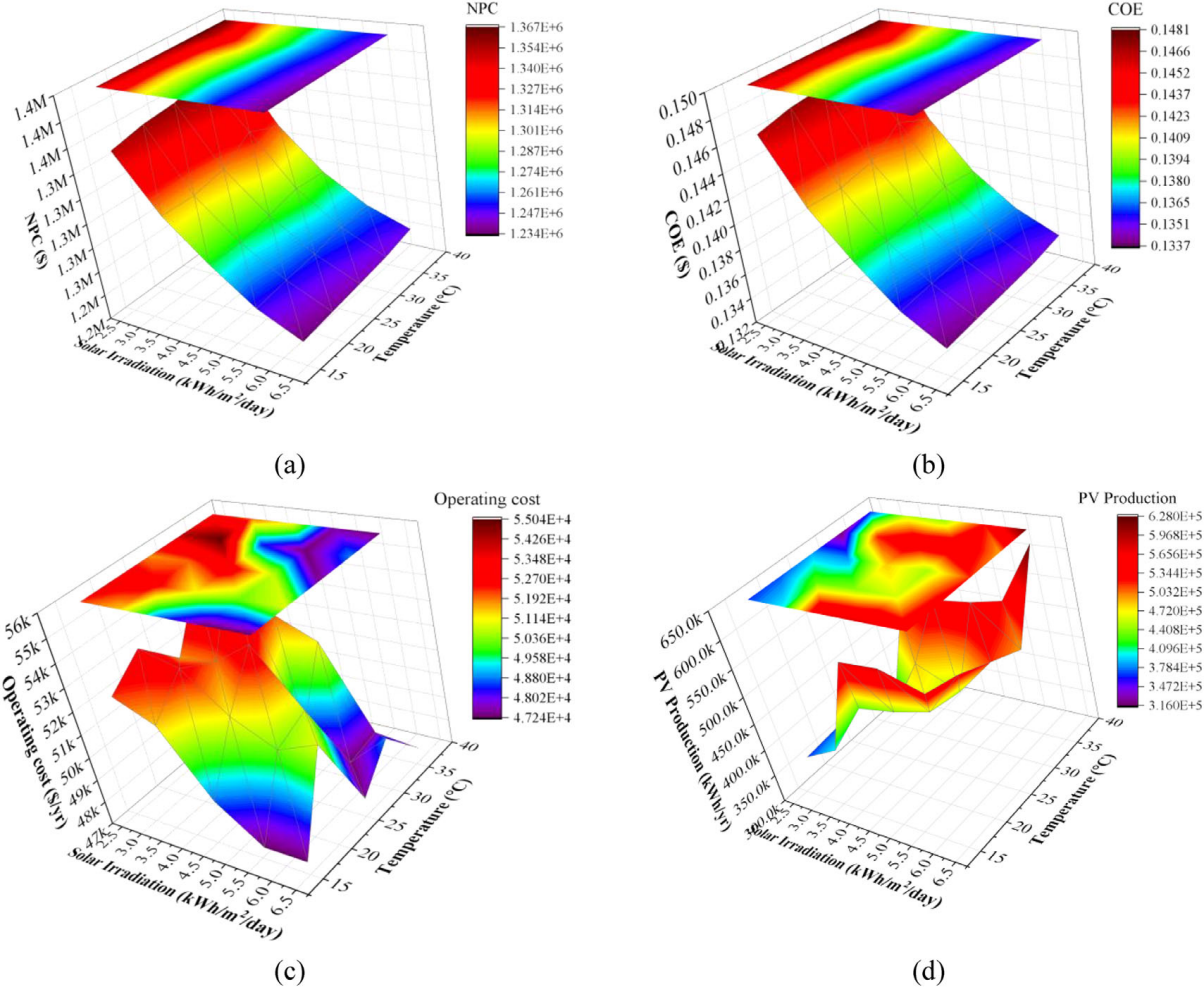
Sensitivity of solar irradiation and ambient temperature on system performance: (a) NPC, (b) COE, (c) Operating Cost, and (d) Annual PV production.

Figure 23 illustrates the sensitivity of the system's techno‐economic performance to variations in solar GHI and ambient temperature. Figure 23a shows that the NPC decreases substantially with increasing GHI, confirming the strong dependence of system economics on solar resource availability. At lower irradiation levels (≈2.5–3.0 kWh/m^2^/day), the NPC reaches about $1.36 million, while at higher irradiation levels (≈6.5 kWh/m^2^/day) it decreases to nearly $1.23 million. Figure 23b presents a similar trend for the COE, which declines from approximately 0.148$/kWh to about 0.134$/kWh as GHI increases. Figure 23c indicates that operating costs are moderately reduced with higher irradiation, reflecting lower reliance on auxiliary energy sources. Figure 23d shows that annual PV electricity production increases markedly with GHI, reaching values above 0.62 GWh at high irradiation levels. In contrast, temperature variations at a fixed GHI have a comparatively minor effect, causing slight increases in NPC and COE and small reductions in PV output due to thermal losses. Overall, the results confirm that solar irradiation is the dominant factor influencing both economic and energy performance, while temperature mainly affects PV efficiency with secondary economic impacts.

**FIGURE 24:**
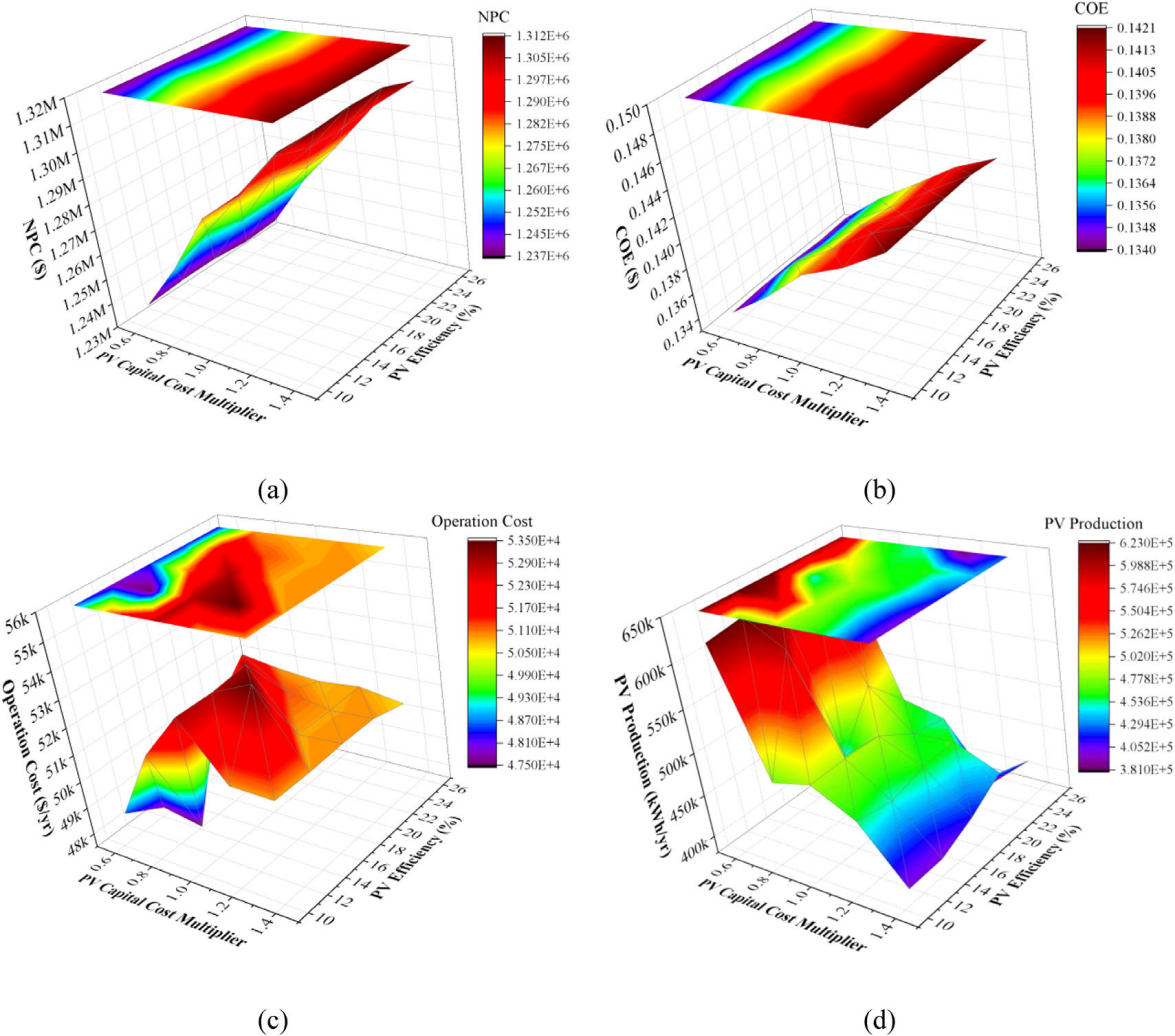
Sensitivity of PV capital cost multiplier and PV efficiency on system performance: (a) NPC, (b) COE, (c) Operating Cost, and (d) annual PV production.

Figure 24 illustrates the sensitivity of system techno‐economic performance to variations in the PV capital cost multiplier and PV module efficiency. Figure 24a indicates that the NPC increases with higher PV capital cost multipliers, while improved PV efficiency mitigates this rise by increasing useful energy yield per installed capacity. Across the explored range, NPC varies from approximately $1.24 million at favorable conditions to about $1.31 million at unfavorable conditions (high cost multiplier/low efficiency). Figure 24b shows a consistent response for the COE, increasing from about 0.134$/kWh to nearly 0.142$/kWh as PV cost multipliers rise, whereas higher efficiencies shift the COE downward due to greater annual generation. Figure 24c demonstrates that operating cost is moderately sensitive to both parameters, reaching roughly 4.75 × 10^4^ $/yr under high‐efficiency conditions and increasing toward about 5.35 × 10^4^ $/yr when efficiency is reduced and PV costs are higher, reflecting greater dependence on non‐PV supply. Figure 24d confirms that PV production is strongly driven by efficiency, increasing from approximately 3.8 × 10^5^ kWh/yr at low efficiency to about 5.8 × 10^5^ kWh/yr at higher efficiency, with secondary influence from the cost multiplier through optimal sizing decisions. Overall, PV efficiency governs energy output, while PV capital cost primarily dictates economic performance.

### 3.4 Correlation of Solar, Wind, Hydrogen, and Battery Performance Indicators

**FIGURE 25:**
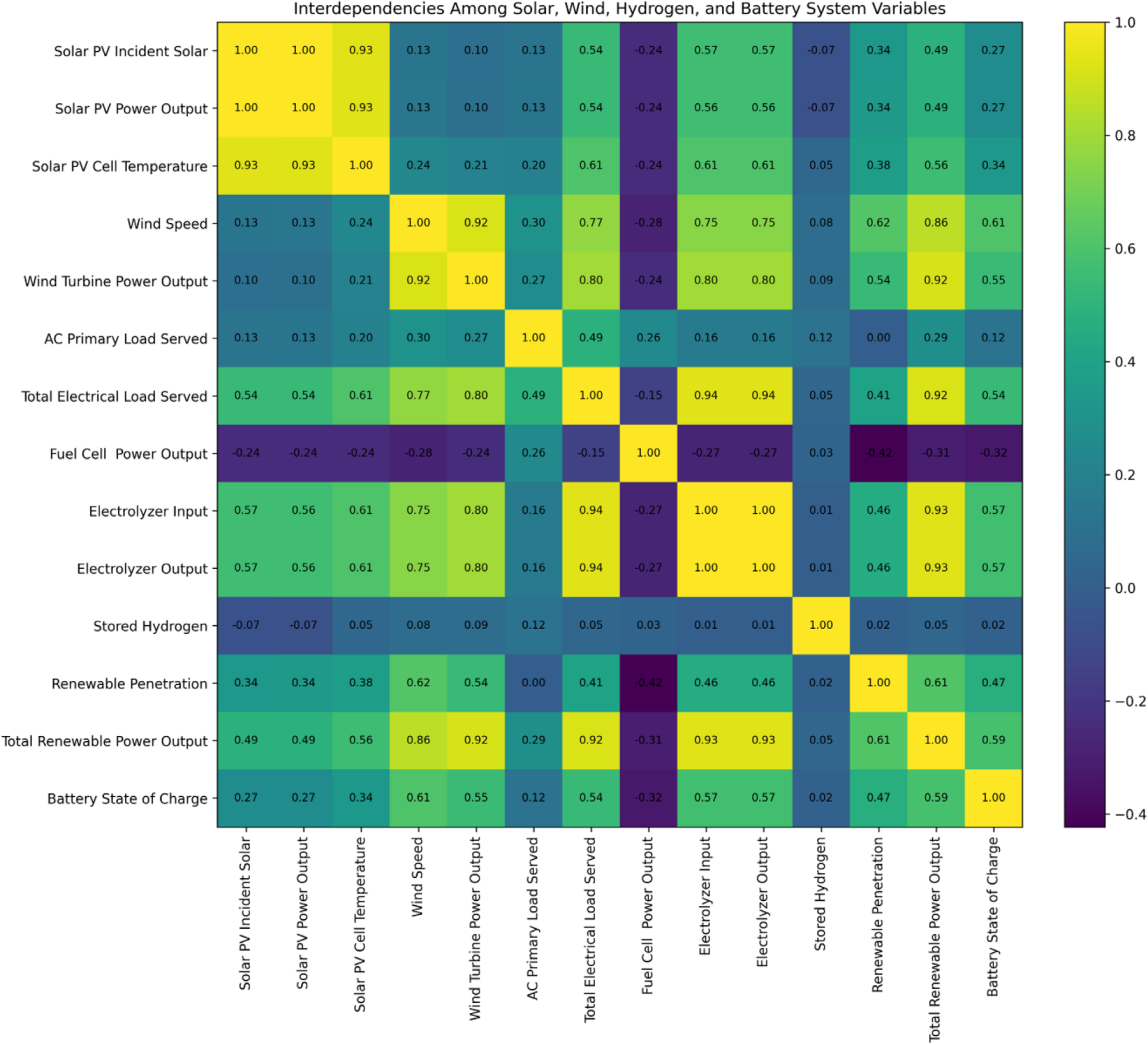
Interdependencies Among Solar, Wind, Hydrogen, and Battery System Variables.

The correlation chart in Figure 25 illustrates the interdependencies among key variables of the integrated solar–wind–hydrogen–battery energy system using Pearson correlation coefficients ranging from −1 to +1. A strong positive relationship is observed between solar PV incident irradiation and PV power output, with a correlation value close to 1.00, confirming that PV generation is directly governed by solar resource availability. Solar PV cell temperature is also highly correlated with irradiation and PV output, with values around 0.93, indicating the thermal response of PV modules under increased solar exposure. Wind speed shows a very strong correlation with WT power output, with a coefficient of about 0.92, highlighting wind speed as the dominant driver of wind energy generation. Total electrical load served exhibits strong positive correlations with electrolyzer input and electrolyzer output, both around 0.94, demonstrating that surplus renewable electricity is largely directed toward hydrogen production. Total renewable power output is also strongly correlated with WT output and electrolyzer operation, with correlation values exceeding 0.90, underscoring the critical role of wind energy in supplying excess renewable power. Renewable penetration displays moderate positive correlations with wind speed, battery SOC, and total renewable power output, indicating that both resource availability and energy storage contribute to higher renewable shares. In contrast, FC power output shows negative correlations with renewable penetration and total renewable power output, confirming its operation as a backup source during periods of low renewable generation. Stored hydrogen exhibits weak correlations with most variables, reflecting its role as long‐term energy storage rather than an immediately responsive component within the system.

### 3.5 Emission Comparison

**FIGURE 26:**
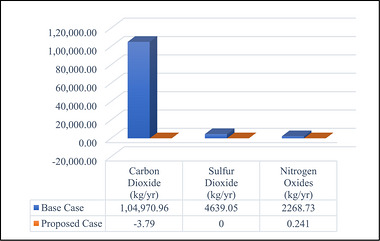
Comparison of emissions of the proposed microgrid with the grid‐only system.

Figure 26 compares annual atmospheric emissions from a grid‐only (base case) system and the proposed microgrid. In the base case, carbon dioxide (CO_2_) emissions are dominant at 104,970.96 kg/yr, while sulfur dioxide (SO_2_) and nitrogen oxides (NO_x_) amount to 4,639.05 kg/yr and 2,268.73 kg/yr, respectively. These values reflect the heavy reliance on centralized fossil‐fuel‐based grid electricity. In contrast, the proposed microgrid demonstrates a drastic reduction in all pollutants. CO_2_ emissions are reduced to –3.79 kg/yr, indicating net carbon neutrality or slight net sequestration due to high penetration of renewable generation and reduced grid imports. SO_2_ emissions are completely eliminated, and NO_x_ emissions are reduced to a negligible 0.241 kg/yr. Overall, the figure highlights the substantial environmental benefits of the proposed microgrid, showing near‐complete mitigation of conventional air pollutants and a transformative reduction in greenhouse gas emissions compared with the grid‐only system.

It is important to clarify the physical interpretation of the reported net CO_2_ emission value of –3.79 kg/year for Case A. In HOMER Pro, emission calculations are performed relative to a reference grid emission factor baseline (kg CO_2_/kWh), which represents the carbon intensity of the conventional electricity source that the proposed microgrid displaces. When the renewable fraction approaches 100%, as in Case A, the system's direct operational CO_2_ output is effectively near zero. The slightly negative net value arises from HOMER Pro's internal emission offset accounting framework: excess renewable electricity generated beyond the community's load demand is credited against the baseline emission factor, producing a marginally negative net figure. This does not imply physical carbon sequestration or active removal of CO_2_ from the atmosphere. Rather, it is an artifact of the software's comparative emission accounting methodology, reflecting the near‐complete avoidance of fossil‐fuel‐derived electricity relative to the assumed baseline. This distinction is consistent with established practices in HOMER‐based microgrid studies and should be interpreted as evidence of outstanding environmental performance, namely, near‐complete decarbonization of the community's electricity supply, rather than literal negative emissions. To contextualize the emission reduction results within a formal carbon accounting framework, it is important to define the system boundary and baseline clearly. The carbon footprint boundary of the proposed microgrid encompasses Scope 1 operational emissions from system components (effectively near‐zero given 100% renewable generation) and excludes upstream manufacturing and end‐of‐life emissions, consistent with the operational boundary convention used in HOMER Pro simulations. The baseline scenario is defined as the counterfactual condition in which the community relies entirely on centralized fossil‐fuel‐based grid electricity, characterized by Bangladesh's national grid emission factor of approximately 0.6823 kg CO_2_/kWh. Against this baseline, the proposed microgrid generates annual avoided emissions of approximately 104,967 kg CO_2_/year, as reflected in the comparison presented in Figure 26. These emission reductions carry significant potential for conversion into tradable carbon assets under internationally recognized voluntary carbon market mechanisms. Under the Verified Carbon Standard (VCS) framework, now administered as Verra's VCS Program, the proposed project could qualify under methodologies such as VM0038 (Methodology for Electric Vehicle Charging Systems) or more appropriately AMS‐I.A and AMS‐I.D under the Clean Development Mechanism (CDM), which cover renewable electricity generation for captive use and grid displacement. For off‐grid rural electrification projects in least‐developed countries such as Bangladesh, the Programme of Activities (PoA) mechanism under CDM/PACM offers a particularly suitable pathway, allowing aggregation of multiple small‐scale microgrid installations under a single coordinating entity, thereby reducing transaction costs and improving financial viability.

Additionally, a prerequisite for carbon credit issuance can be demonstrated through a barrier analysis, showing that the project would not be financially viable without carbon revenue, given its high capital expenditure (CAPEX of $594,206 for Case A), and through an investment analysis, confirming that the internal rate of return falls below the benchmark without carbon credits. At a conservative carbon price of $10–$15 per tonne of CO_2_ (consistent with current voluntary market rates for Least Developed Country projects), the annual avoided emissions of approximately 104.97 tCO_2_/year could generate carbon revenues of $1,050–$1,575 per year. Over the 25‐year project lifetime, this corresponds to a cumulative carbon revenue of approximately $26,250–$39,375, which, when discounted and subtracted from the NPC, could reduce the effective NPC by approximately 2–3%, improving project economics and lowering the effective COE. While this represents a modest but non‐negligible improvement, it establishes an important precedent for integrating carbon finance into the techno‐economic assessment of off‐grid renewable microgrids in Bangladesh and similar developing‐country contexts, potentially catalyzing broader deployment through blended finance mechanisms.

### 3.6 Comparison with Other Published Works

Table 9 presents a comparative analysis between the outcomes of the proposed study and those reported in existing literature. The comparison focuses on key technological configurations and economic indicators to highlight similarities and differences in system performance and cost characteristics across the reviewed studies. This comparative evaluation serves to substantiate the methodology adopted in the present work and situates its findings within the wider context of renewable energy system research, thereby enhancing the credibility and relevance of the proposed approach.

**TABLE 9:** Comparison of the proposed work with other published works.

System Structure	Location	System type & Category	RF (%)	NPC ($) and COE ($/kWh)
PV‐EL‐HT‐FC [74]	Oman	― community	100	317,401,965 $0.97
PV‐WT‐FC [75]	Abha, Saudi Arabia	Off grid Residential Microgrid	100	75,428 1.208
PV‐WT‐BESS [76]	Aswan City, Egypt	Off‐grid City	—	853,635 0.255
PV‐WT‐DG‐BESS [77]	Maharashtra, India	Off‐grid Village	—	569,275 0.157
PV–WT–BESS–Electrolyzer H_2_ station [78]	Gökçeada Island, Turkey	Off Grid ―	100	8,429,124 1.90
PV‐WT‐BESS [76]	Aswan City, Egypt	Off‐grid City	—	853,635 0.255
PV‐WT‐DG‐BESS [79]	Hatiya, Bangladesh	Off‐grid Village	61.6	1,128,095 0.183
PV‐DG‐BESS [80]	Bilgo, Burkina Faso	Off‐grid Village	29.7	1,177,376 0.524
PV‐DG‐BESS [81]	Malaysia	Off‐grid Village	60	425,000 1.61
PV‐BESS‐DG [82]	Ladakh, India	Off‐grid Village	—	278,176 0.29
PV‐WT‐BESS‐DG [83]	North Pyongan, North Korea	Off‐grid Village	—	472,719 0.246593
PV‐DG‐BESS [84]	Bellavista, Ecuador	Off‐grid Community	22.7	102,027 0.559
PV‐DG‐BESS [85]	Kuakata, Bangladesh	Off‐grid Village	61.79	5,190,000 0.367
PV‐DG‐BESS [86]	Gyeonggi Province, South Korea	Off‐grid Sejong Academy, Suwon	76	1,615,112 0.673
The Proposed Work	Char Ishwar, Bangladesh	Off‐grid Community	100	1,279,908 0.139

## 4 Conclusion

This study presents a comprehensive techno‐economic and environmental assessment of an off‐grid hybrid renewable microgrid designed for rural electrification in Char Ishwar, a coastal union of Noakhali District, Bangladesh. Using HOMER Pro software, several system configurations integrating solar PV, WT, BESS, electrolyzers, H_2_ storage tanks, and FCs were evaluated under realistic load and resource conditions. Among the three scenarios analyzed, the PV–WT–BESS–Electrolyzer–FC–H_2_ Tank configuration (Case A) emerged as the optimal solution. Case A achieved the lowest NPC of approximately $1.28 million, although its practical deployment requires supportive financing mechanisms to address high upfront capital costs and a competitive COE of about $0.139/kWh, while reliably supplying electricity to 180 rural households. The complementary use of solar and wind resources, supported by short‐term battery storage and long‐term hydrogen energy storage, effectively mitigates renewable intermittency and ensures continuous power availability. From an environmental perspective, the system demonstrates outstanding performance by achieving net negative CO_2_ emissions of −3.79 kg/year, underscoring the importance of green hydrogen in the deep decarbonization of off‐grid energy systems. LCOH was estimated at $6.57/kg, indicating economic feasibility when optimally integrated with renewables and batteries. Sensitivity analysis identifies wind speed and electrical load as the most influential parameters affecting system economics. Although the proposed optimal configuration (Case A) demonstrates strong techno‐economic performance, its initial capital cost of approximately $594,206 may appear high when distributed across 180 rural households. However, in the context of rural Bangladesh, such infrastructure projects are typically not financed solely by individual users. Instead, successful deployment models, such as those implemented under the IDCOL program, rely on a combination of government subsidies, concessional loans, international donor support, and public–private partnerships. These financing mechanisms significantly reduce the upfront burden on households while enabling long‐term cost recovery through affordable tariff structures or microcredit schemes. Furthermore, community‐based ownership models and pay‐as‐you‐go systems can enhance accessibility and financial sustainability. Therefore, while the initial investment is substantial, the proposed microgrid remains economically feasible when supported by appropriate policy frameworks, financial instruments, and institutional arrangements tailored to rural electrification in developing regions. Overall, the findings confirm that hydrogen‐integrated hybrid renewable microgrids offer a reliable, sustainable, and cost‐effective electrification pathway for remote coastal regions of Bangladesh.

## Funding

This work was supported by the Deanship of Scientific Research, Vice Presidency for Graduate Studies and Scientific Research, King Faisal University, Saudi Arabia [Grant No. KFU262127].

## Conflicts of Interest

The authors declare no conflicts of interest.

## Data Availability

Data will be available upon request from the corresponding author.
